# Surface-Enhanced Spatially Offset Raman Spectroscopy in Tissue

**DOI:** 10.3390/bios14020081

**Published:** 2024-02-02

**Authors:** Dayle Kotturi, Sureyya Paterson, Mike McShane

**Affiliations:** 1 Department of Biomedical Engineering, Texas A&M University, College Station, TX 77843, USAsureyya.paterson@me.com (S.P.); 2 Department of Materials Science and Engineering, Texas A&M University, College Station, TX 77843, USA

**Keywords:** surface-enhanced spatially offset Raman spectroscopy, SESORS, implantable, biocompatible, hydrogel, Monte Carlo modeling, light-tissue interaction, tissue optics

## Abstract

One aim of personalized medicine is to use continuous or on-demand monitoring of metabolites to adjust prescription dosages in real time. Surface-enhanced spatially offset Raman spectroscopy (SESORS) is an optical technique capable of detecting surface-enhanced Raman spectroscopy (SERS)-active targets under a barrier, which may enable frequent metabolite monitoring. Here we investigate how the intensity of the signal from SERS-active material varies spatially through tissue, both experimentally and in a computational model. Implant-sized, SERS-active hydrogel was placed under different thicknesses of contiguous tissue. Emission spectra were collected at the air-tissue boundary over a range of offsets from the excitation site. New features were added to the Monte Carlo light-tissue interaction model to modify the optical properties after inelastic scattering and to calculate the distribution of photons as they exit the model. The Raman signals were detectable through all barrier thicknesses, with strongest emission for the case of 0 mm offset between the excitation and detector. A steep decline in the signal intensities occurred for offsets greater than 2 mm. These results did not match published SORS work (where targets were much larger than an implant). However, the model and experimental results agree in showing the greatest intensities at 0 mm offset and a steep gradient in the intensities with increasing offset. Also, the model showed an increase in the number of photons when the new, longer wavelengths were used following the Stokes shift for scattering and the graphical display of the exiting photons was helpful in the determination and confirmation of the optimal offset.

## 1. Introduction

The basis of Raman spectroscopy (RS) is that when light interacts with a substance, a small portion of the scattered light changes wavelength, based on the vibrational pattern of certain chemical bonds. The resultant spectra can be used to identify the substance [1]. The practical usefulness of this phenomenon increases significantly when the intensity of the inelastic scattering is amplified via the addition of pure metals which create surface plasmon resonance. This technique is called surface-enhanced Raman spectroscopy (SERS) [2]. RS was further adapted to a technique called spatially offset Raman spectroscopy (SORS) where, by offsetting the detection point from the excitation point, the identity of molecules below the surface or through a membrane can be detected. SORS makes use of the behavior of the random walk of a photon in 3D space. It is statistically more likely for a photon that has gone deep into the material to exit the material further away. SORS was introduced as an idea in 2005 and experimentally demonstrated in 2010 [3,4]. When SERS-active targets are used with a SORS detection setup, the two techniques are combined to perform surface-enhanced spatially offset Raman spectroscopy (SESORS).

**Figure 1:**
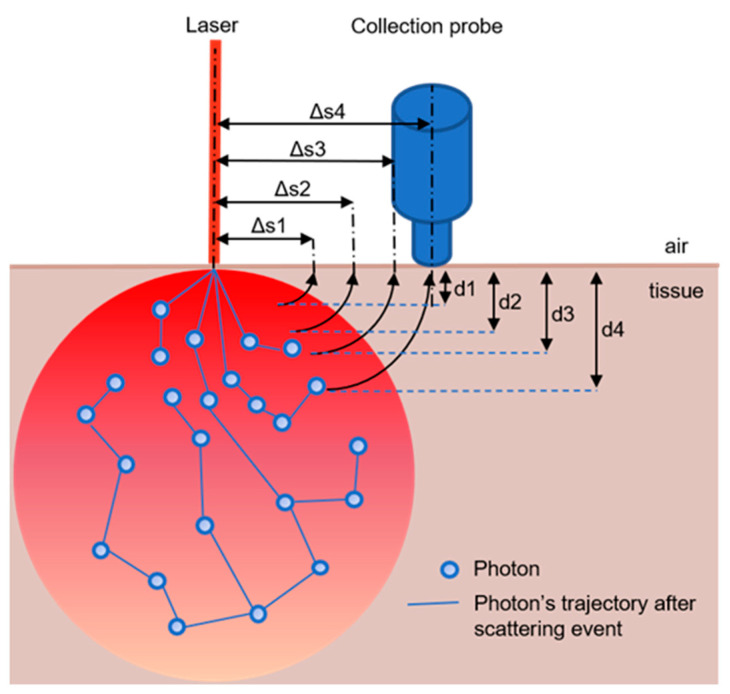
SORS geometry where *s* = offset and *d* = depth. Scattering signal is collected from deeper in the sample as Δ*s* increases. Adapted from [17].

While SORS has been established as a reliable technique for verifying food purity in agriculture and detecting dangerous substances at airports [5,6], it is still in development as a technique to detect chemical levels under the skin in biosensing [3,4,7,8,9,10,11,12,13,14,15]. As mentioned above, SORS works by non-invasively detecting the presence and concentration of chemical signals of interest through a barrier. In some studies, the RS signals are measured repeatedly as the separation between the excitation laser and the detector’s collection point is increased (Figure 1) [9,16]. In other studies, a single, fixed offset is used, usually due to a limitation of the instrument [8,13,14]. SORS results typically show that the intensity of the RS signals of the target increases and then declines as the offset increases, while the intensity of the RS signal of the barrier (if there is one) steadily decreases as the offset increases. The offset associated with the maximum target signal depends on the depth of the target under the surface and the optical properties of the turbid media.

Results from SORS studies provide some very encouraging findings that demonstrate potential useful application by improving the SNR of measurements through turbid media. First, the intensity of a 2 mm layer of trans-stilbene powder target at 1 mm depth under PMMA (poly(methyl methacrylate)) was measured to be 19 times stronger when the offset is 3.5 mm than when the offset is 0.0 mm [3]. Second, conventional RS was unable to detect the target through a 1.1 mm thick container wall while, using SORS with a 10 mm offset, the characteristic peak of hydrogen peroxide at 876 cm^−1^ can be detected [10]. Third, the 1,2-bis(4-pyridyl)ethylene (BPE) and 4-(1H-pyrazol-4-yl)pyridine (PPY) nanotags were detectable in pork up to 60 mm deep using a handheld SORS spectrometer with an 8 mm offset [8].

Most biomedical SORS studies have been performed on the benchtop. Often the authors claim that the results extrapolate to be of potential use in vivo, but for several reasons their specific experimental setups are limited in their applicability to likely real-world scenarios. For example, the detector is sometimes placed on the opposite side of the excitation (TRS, a.k.a. 180-degree SORS). A study used TRS to detect SERS nanoparticles (NPs) buried in 25 mm mammalian tissue at different concentrations [4]. Another used TRS to detect SERS signals through a 14 cm thickness of porcine tissue with a laser exposure less than the maximum allowed [18]. While this is important for assessing deep tumors, the detection of more superficial implants is less invasive when approached using the SORS setup. Since emitted photons are collected on the same side of the body as the laser excitation, the SORS configuration is independent of the thickness of the anatomy. Second, the target is much larger than an actual implantable (injectable) device. Often the target is the same size as the barrier layer material or the entire sensing area [3,9]. Having a target which covers the entire sensing area (e.g., 25 × 75 mm) is not likely to translate to the in vivo case of an implant. Typically-implanted devices are substantially smaller to facilitate in vivo deployment and limit tissue trauma; in fact, most in vivo implants are actually targeted for needle insertion so as to avoid a surgical procedure. As an example of practical dimensions, an 18-gauge needle limits the implant to 0.838 mm in diameter. Third, the barrier layer used in laboratory tests may consist of a stack of discrete layers rather than contiguous material [8,9,19]. There is a refractory boundary for the photons between each layer in the stack which reduces the photon’s path length compared to traveling through a single contiguous layer. Fourth, there is no tissue-mimicking material under the target where the interaction occurs, so the photons cannot propagate there as part of their random walk before scattering laterally and upwardly [9]. In the in vivo case, there would be turbid media under the implant as well, which would provide the photons increased scattering opportunities back to the surface.

Regarding instrumentation, several studies show setups suitable for in vivo measurements [7,9,11]. An example setup is shown in Figure 1. However, only one published paper has actually used this configuration for animal studies [9]. This study completed only a single set of in vivo measurements prior to sacrifice and therefore was unconcerned with the in vivo requirement of long-term usefulness and the effects of the foreign body response, inflammation, migration of the SERS-active target in the body and toxicity to other organs. In fact, this study relied on the migration of the NPs in the body because it injected them into the tail vein (rat model) and detected them in the skull. Therefore, it should be emphasized that the only in vivo SORS detection known to have been successfully completed is still far from demonstrating the ability of the SERS-active target to perform as a long-term implanted biosensor.

SORS lends itself well to Monte Carlo modeling because the interaction of light in turbid media (such as tissue) is well understood and because the phenomenon of Raman scattering can be expressed as a probability. Early efforts to model RS with Monte Carlo simulations occurred in 1995 and 2004 [20,21]. These first works did not vary the offset between the excitation and detection sites. Rather, they showed that by lagging the time of detection (gating), it was possible to collect photons that went deeper in the 3D space. Mosca et al. [19] modeled how deep photons are able to go below the surface in an effort to predict the offset required to detect a target at a specific depth. Compared to empirical results, the numerical simulations matched well for the spatial offsets of 0 and 5 mm. Even though the match was not as good in the 10 mm offset case, it is the only case where the SORS intensity under the surface exceeds the intensity at the surface when the target is 4 mm under the surface and the offset is 10 mm.

This study makes several unique contributions to SORS research, especially related to its usefulness for in vivo sensing of implanted SERS-active targets. First, this study improves upon several limitations found in other studies, thereby moving SORS closer to being a CM tool used for in vivo biosensing. These improvements consist of: reducing the target to the size of an implant rather than being as extensive in length and width (in the plane parallel to the sensing surface) as the barrier layer; using a solid tissue barrier layer on top of the target rather than a stratified barrier; and providing a base under the target to add to the traversable volume for the photons’ random walk. Second, this study extends the modeling of SORS by using dynamic optical properties that change when inelastic scattering occurs. Therefore, unlike previous RS modeling, the scattering length is no longer constant over the simulation. This study also adds a graphical representation (heatmap) of the final position of the photons as they leave the model, which is key in evaluating the optimal placement of the detector.

## 2. Materials and Methods

The materials and methods used in this study closely match those reported in [22] and therefore only the differences are listed here.

### 2.1. Instrumentation

#### Raman Spectroscopy

One dark spectrum and fifteen raw spectra were collected at each offset. The dark was subtracted from each raw spectrum and cosmic rays of ≤2 wavenumbers in breadth and ≥15% of the range of intensities were removed prior to baseline correction. While 15% is a relatively small percentage, the restriction on the breadth of the spike was sufficient to correctly distinguish cosmic arrays from actual MBA reference peaks (which typically span 20 wavenumbers). Baseline correction was accomplished via an asymmetric least-squares method [23].

### 2.2. Methods

#### 2.2.1. Porcine Muscle Tissue

A large slab of cured pork was purchased from a local supermarket. It was sliced into a range of thicknesses from 1 to 5 mm using a 200-W electric adjustable-thickness meat slicer by OSTBA.

#### 2.2.2. SORS Acquisition

**Figure 2:**
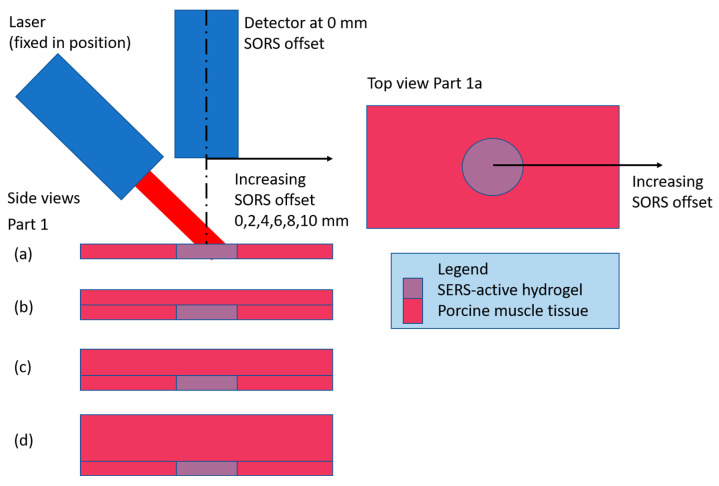
Experimental setup for Part 1. Parts 1a–d show the barrier thickness increasing from 0 to 3 mm, respectively, in 1 mm increments. Parts 2–5 are variations of this setup as described in Table 1.

The SORS experimental setups consist of Parts 1–5 (Table 1). All parts use a 6 mm diameter, 1 mm thick disc of SERS-active pHEMA (target), first uncovered and measured with detector at a 0 mm SORS offset and then through different thicknesses of porcine muscle tissue and measured with detector at a range of SORS offsets and step size. Figure 2 is a diagram of the setup for Part 1a–d. As detailed in Table 1, the setups for Parts 2–5 are variations of Figure 2 with the addition of a base layer of porcine tissue under the target as well as using contiguous vs. stratified porcine tissue on top of the target.

#### 2.2.3. Modeling

**Figure 3:**
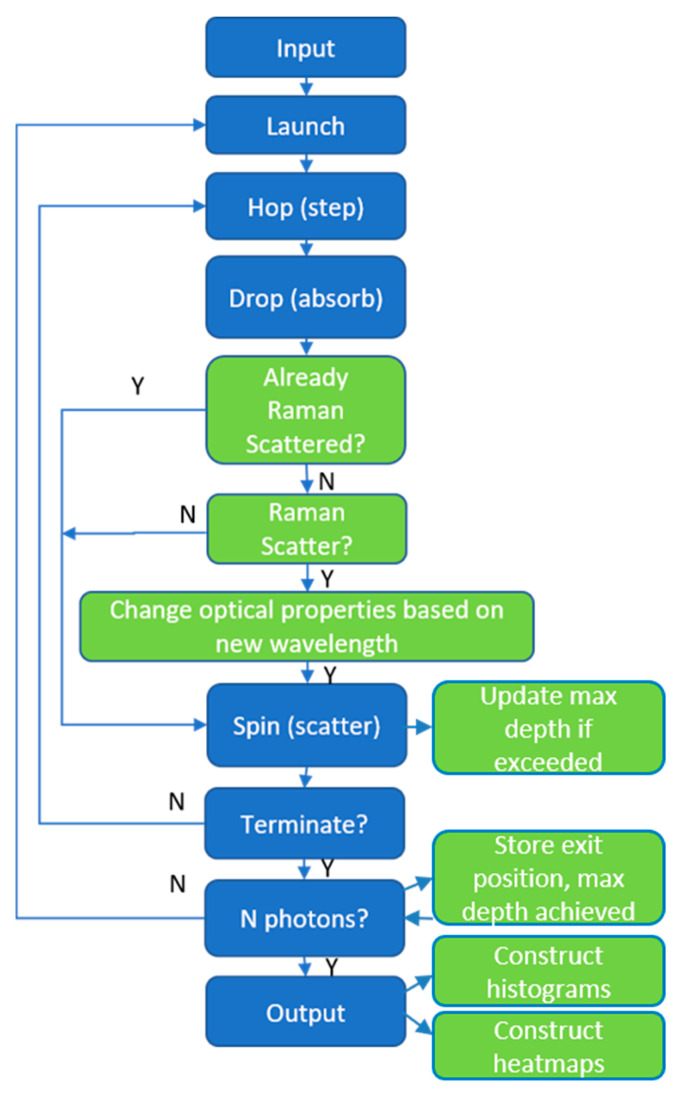
The original modeling steps are shown in blue [24]. The steps added in this work to incorporate inelastic scattering and modifications to optical properties are shown in green. Y = yes and N = no.

The Oregon Medical Laser Center (OMLC.org) has made their light-tissue modeling software publicly available for more than twenty years [24]. The software at https://omlc.org/software/mc/mcml/index.html was downloaded on 21 October 2019 and modified to add inelastic scattering events based on a probability and to update the optical parameters as a function of the new wavelength (Figure 3).

A detailed description of the modifications to the software, including the optical properties used by tissue type and wavelength, is provided in the Supplementary Information. Briefly, given a scattering event, the probability that it is an inelastic or Raman scattering event is set to 1 × 10^−2^ in the model. This is artificially high in order to adapt the Monte Carlo method to the weak process, but this approach has been established by others [21,25]. This probability was deemed acceptable given the goal to gain understanding of the spatial distribution of the photons when they exit the upper surface of the model and not to quantitatively predict the exiting fluence.

**Figure 4:**
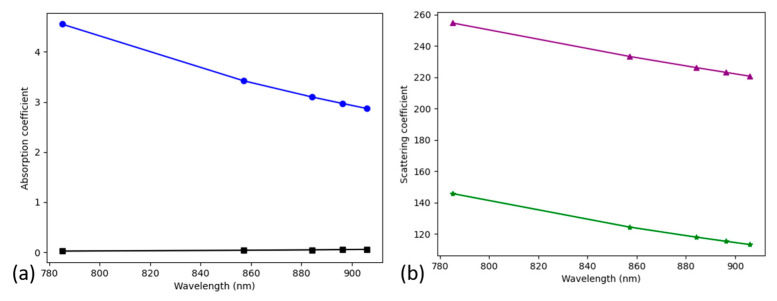
Optical properties: (**a**) absorption coefficient, µ_a_, for epidermis (blue circles) and dermis (black squares) and (**b**) scattering coefficient, µ_s_, for epidermis (purple triangles) and dermis (green stars).

**Figure 5:**
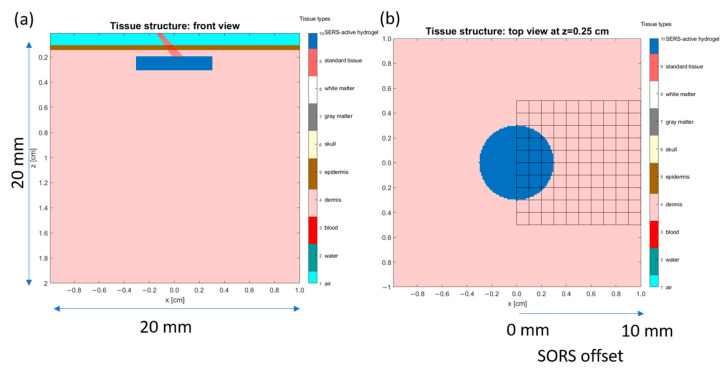
Model of tissue and implanted SERS-active target used in the Monte Carlo simulations. (**a**) Front (cross-sectional) view. (**b**) Top view at depth z = 0.25 cm. Grid shows location of the heatmap (in subsequent discussion).

The new wavelengths of the inelastically scattered photons are based on a second set of probabilities, associated with the area under the curve (spectrum) at the peaks of interest. The optical parameters for all the wavelengths of interest are calculated for all tissue types at the start and stored in a 2D matrix. After a photon has changed wavelength, the optical properties used during the remainder of its trajectory through the structure are changed to those of the new wavelength. Plots of the optical properties as a function of wavelength are shown in Figure 4a,b, for absorption coefficient, µ_a_, and scattering coefficient, µ_s_, respectively. The epidermis and dermis are shown since they are the most relevant tissue types for the model (Figure 5). It is hypothesized that, as far as Raman scattering is concerned, the decreasing values of µ_s_, which are inversely related to the scattering distance, will cause an increase in the amount of emitted light from the model, since the photon travels further in each step of its path.

To model the detection of photons at different offsets, the exiting surface is divided up into a grid of 1 mm squares. The final photon position was assigned to the square (“bin”) that contained its (x, y) position at exit. The 2 × 2 × 2 cm tissue model is shown in Figure 5. It includes the SERS-active hydrogel disc location and the laser position. The laser is shown to be aligned to be centered on the disc, even though the photons will scatter upon entering the tissue. The excitation beam was modeled as a point source at x = y = z = 0 propagating in the direction of 45 degrees from the vertical (to match the experimental setup). By setting the launch position and direction, the software does not perform a convolution to create the broad beam response. The result is a beam width which could be narrower than the experimental case; this could cause an underestimate in the size of the scattering volume used by the photons.

## 3. Results

### 3.1. Experimental

A set of experiments were conducted where a 6 mm diameter disc of SERS-active hydrogel (target) was placed under various thicknesses of contiguous porcine muscle tissue, with and without a porcine muscle tissue base. Raman spectra were collected with the detector positioned at a range of offsets from the target to characterize the signal intensity with detector offset. To quantify the losses incurred by the addition of the barrier, spectra were also collected with the hydrogel uncovered (and the detector was positioned at a 0 mm offset). The results of the experiment are shown in plots of the intensities of four characteristic MBA peaks (the SERS-active material) vs. detector offset, for each thickness of barrier (subplots a–d or e). The generic configuration for all parts of the experiment is drawn in Figure 2, with the variations described in Table 1. The first four parts of the experiment build on each other to find the optimum setup (adding the base layer, optimizing the offset step size and adding a 4 mm thick barrier). Due to the repetition in the first four parts, plots for Parts 1–3 are in the Supplementary Information and Part 4 results are presented here. Part 5 is a comparison case for Part 4 to evaluate the effect of a solid (contiguous) barrier over the target vs. a layered (stratified) barrier. The results of Parts 4 and 5 are described in Section 3.1.1, Section 3.1.2 and Section 3.1.3).

#### 3.1.1. Contiguous Barrier Case: 1, 2, 3 and 4 mm Thick Barrier Layers on 5 mm Thick Porcine Muscle Tissue Base, 0 through 5 mm Offsets in 1 mm Steps

**Figure 6:**
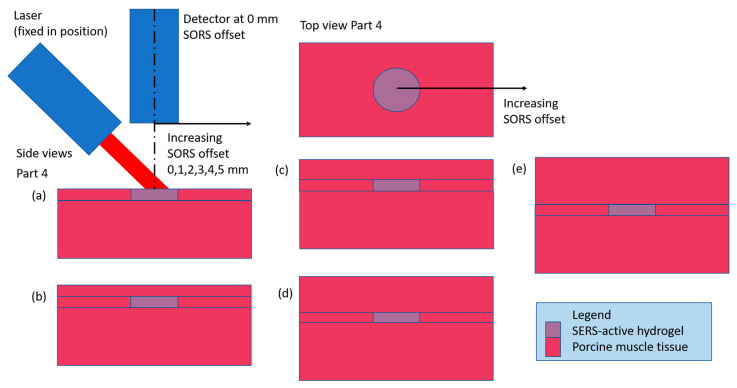
Part 4 setup: all barrier thicknesses above the target SERS-active hydrogel are contiguous. The labels (**a**–**e**) correspond to the five different barrier thicknesses, 0 to 4 mm, respectively, in 1 mm increments.

**Figure 7:**
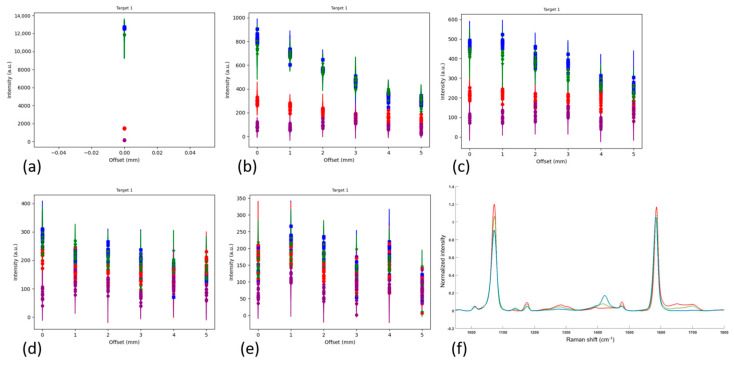
Raman scattering intensities of hydrogel discs as measured in five different experimental setups. (**a**) uncovered disc, (**b**) disc under 1 mm tissue, (**c**) disc under 2 mm tissue, (**d**) disc under 3 mm tissue, (**e**) disc under 4 mm tissue, all on 5 mm solid porcine muscle tissue base. Legend for (**a**–**e**): blue = 1072 cm^−1^ peak, green = 1582 cm^−1^ peak, red = 1430 cm^−1^ peak, purple = 1702 cm^−1^ peak. N = 15. Circle is mean, error bars show min and max of the 15 values. Integration time = 2000 ms, laser output power = 66 mW. (**f**) Reference spectrum for MBA in hydrogel. Legend for (**f**): red = pH 4, green = pH 7, blue = pH 10 environment.

The experimental setup for the contiguous barrier case (also known as Part 4) is shown in Figure 6. Figure 7a shows the maximum intensity of the two reference peaks (blue and green) to be ~12,000 a.u. When the 1 mm barrier layer is applied (Figure 7b), the signal is degraded to less than 1000 a.u., or ~8% of the maximum for the zero offset. There is a linear decline thereafter as the offset increases to 5 mm. Figure 7c, the 2 mm barrier layer case, shows a slight increase in the 1072 cm^−1^ peak (blue) at an offset = 1 mm compared to zero offset. The 1582 cm^−1^ peak (green) doesn’t follow this trend, however, as some of the intensities at the 1 mm offset are below those at the 0 mm offset. Overall, Figure 7c shows declining intensities with increasing offset over the full range to 5 mm. In Figure 7d, the 3 mm barrier layer, the intensities have degraded by another third and do not show any increase in intensity with increased offset. Finally, in Figure 7e, the new 4 mm thick barrier layer addition, the intensities are similar to those in Figure 7d but the presence of an outlier in some of the 1430 cm^−1^ pH-sensitive peak values is affecting the y-scale. Also, in Figure 7e, the intensity data are more randomly distributed at each offset than in Figure 7a–d, where the reference peaks (blue and green) are highest, followed by the 1430 cm^−1^ pH-sensitive peak (red), followed by the 1702 cm^−1^ pH-sensitive peak (purple). For reference, Figure 7f shows the Raman spectrum of MBA, the Raman-active material in the hydrogel.

#### 3.1.2. Stratified Barrier Case: 1 mm Thick Barrier Layers Combined to Make a 1, 2, 3 and 4 mm Stratified Thickness on a 5 mm Contiguous Base, 0 through 5 mm Offsets in 1 mm Steps

**Figure 8:**
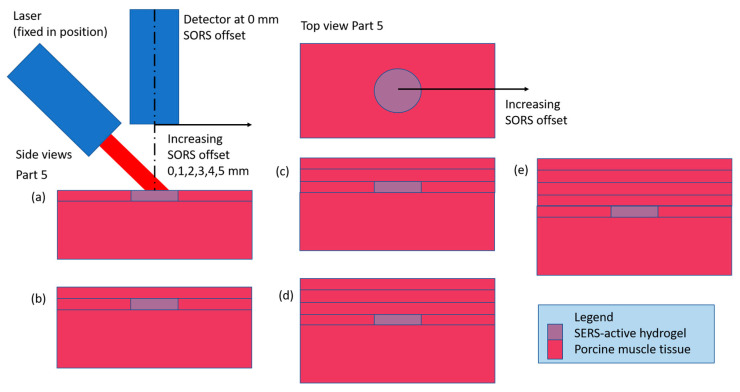
Part 5 setup: all barrier thicknesses above the target SERS-active hydrogel are stratified layers of 1 mm thick barriers. The labels (**a**–**e**) correspond to the five different barrier thicknesses, 0 to 4 mm, respectively, in 1 mm increments.

**Figure 9:**
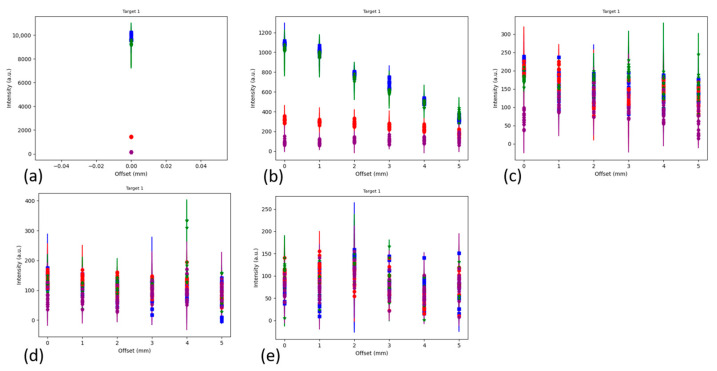
Raman scattering intensities of hydrogel discs as measured in five different experimental setups. (**a**) uncovered disc, (**b**) disc under 1 mm tissue, (**c**) disc under two slices of 1 mm tissue, (**d**) disc under three slices of 1 mm tissue, (**e**) disc under four slices of 1 mm tissue, all on 5 mm solid porcine muscle tissue base. Legend for (**a**–**e**): blue = 1072 cm^–1^ peak, green = 1582 cm^−1^ peak, red = 1430 cm^−1^ peak, purple = 1702 cm^−1^ peak. N = 15. Circle is mean, error bars show min and max of the 15 values. Integration time = 2000 ms, laser output power = 66 mW. (**f**) Reference spectrum for MBA in hydrogel. Legend for (**f**): red = pH 4, green = pH 7, blue = pH 10 environment.

The experimental setup for the stratified barrier case (also known as Part 5) is shown in Figure 8. This case is a comparison dataset to evaluate the effect of a solid (contiguous) barrier over the target vs. a layered (stratified) barrier. Instead of using the barrier layers with a range of thicknesses from 1 to 4 mm, in Part 5 the barrier layers of various thicknesses are created from a stack of 1 mm thick slices. Figure 9a shows the maximum intensity as ~10,000 a.u. for the two reference peaks (blue and green). When the first 1 mm barrier layer is applied (Figure 9b), the signal is degraded to about 1100 a.u., or 11% of the maximum for the zero offset. There is a decline thereafter as the offset increases to 5 mm, although it is more linear for the 1582 cm^−1^ peak (green) than for the 1072 cm^−1^ peak (blue). Once a second 1 mm barrier layer is added (Figure 9c) and beyond (Figure 9d,e), no linear trends are apparent. There are outliers that skew the y-scale, but ignoring them, the intensities are close in magnitude for these three cases: Figure 9c has maximum magnitudes of ~250 a.u., Figure 9d has maximum magnitudes of ~200 a.u. and Figure 9e has maximum magnitudes of ~150 a.u. The data in Figure 9 show that when stratified barrier layers are used over the target, the SORS signals are greatly impeded. This was expected and was in fact a main driver for these experiments. The addition of all the refractory boundaries between the layers was expected to impact the photons’ trajectories and this is the first attempt to quantify the attenuation. For reference, Figure 9f shows the Raman spectrum of MBA, the Raman-active material in the hydrogel.

#### 3.1.3. Comparison of Contiguous and Stratified Barrier Cases

**Figure 10:**
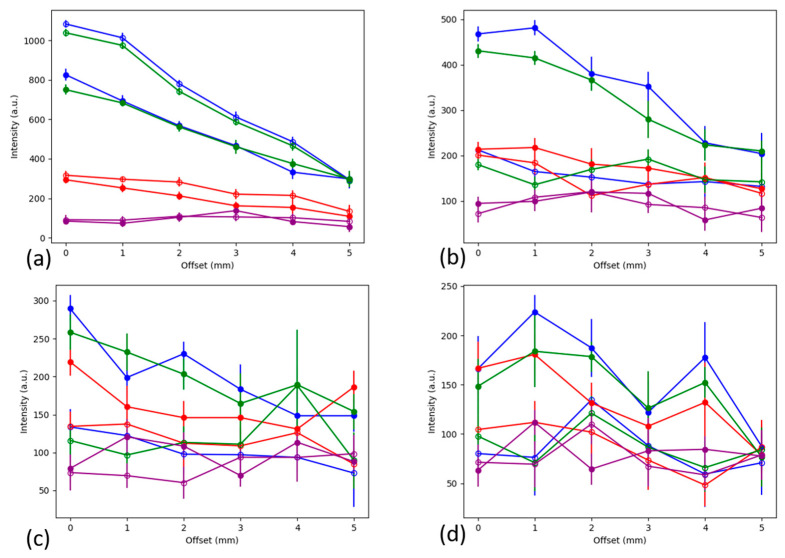
Overlaying the results using the contiguous and stratified barriers for barrier thicknesses of (**a**) 1 mm, (**b**) 2 mm, (**c**) 3 mm and (**d**) 4 mm. The markers indicate the Raman peak measured through the contiguous barrier and stratified barrier, with solid and hollow circles, respectively. Legend: blue = 1072 cm^−1^ peak, green = 1582 cm^−1^ peak, red = 1430 cm^−1^ peak, purple = 1702 cm^−1^ peak.

To better compare the results using the contiguous and stratified barriers, the results have been overlaid in Figure 10. In Figure 10a, the two cases have the same physical setup, both the contiguous (squares) and the stratified (circles) have a 1 mm thick barrier, which means that the signals should be similar in magnitude. Note that the reference peak values (blue and green) for both cases (contiguous and stratified) are much greater than the other peaks (red and purple) and that there is even a stronger signal for the stratified case, owing perhaps to better alignment of the laser and detector with the sample. However, this stronger signal is already lost when the second stratified 1 mm layer is added. In Figure 10b, the reference peak values (blue and green circles) are greatly reduced compared to the 2 mm contiguous layer (blue and green squares). This trend continues as the barrier thicknesses increase in Figure 10c,d.

### 3.2. Monte Carlo Modeling

In a single simulation, approximately two million (1.861 × 10^6^) photons were introduced into the 10 × 10 × 2 mm tissue model space in an elapsed time interval of 11.831 min. Each photon traversed through the space and was either absorbed or scattering at every step based on random events. There were 405,319 inelastic scattering events. The number of times that the new wavelengths matched the array of pH 7.0 target peaks (1072, 1430, 1582 and 1702 cm^−1^) was: 151,883, 43,362, 188,437 and 21,637, respectively. The maximum number of steps taken by any photon was 11,768. While only one Raman event was allowed per photon, the criteria for an additional inelastic event were met 34,823,817 times. The total number of scattering (elastic and inelastic) events was 653,474,740.

**Figure 11:**
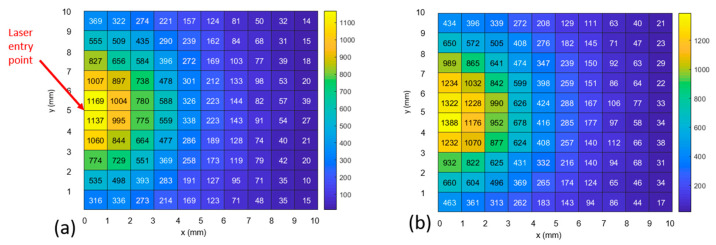
For photons that inelastically scatter to the 1072 cm^−1^ peak, heatmaps show the final photon position in horizontal plane at tissue surface. pH level = 7.0. (**a**) no change to optical parameters after the event. (**b**) with change to optical parameters after the event.

**Figure 12:**
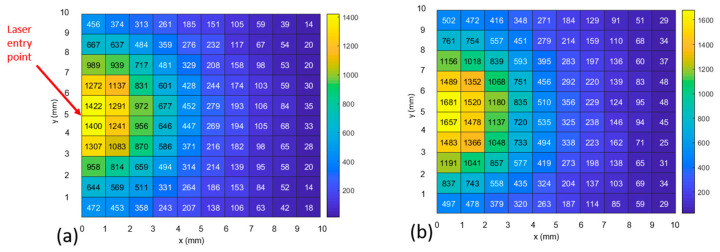
For photons that inelastically scatter to the 1582 cm^−1^ peak, heatmaps show the final photon position in horizontal plane at tissue surface. pH level = 7.0. (**a**) no change to optical parameters after the event. (**b**) with change to optical parameters after the event.

The results of a single, representative Monte Carlo simulation are shown in Figure 11 and Figure 12. Figure 11 shows the case where photons scatter into the 1072 cm^−1^ bin (i.e., they changed their wavelength from the excitation wavelength as a result of an inelastic scattering event). Comparing Figure 11a to Figure 11b, the number of photons exiting the model increases by 13% in the first millimeter from the excitation point to 30% by the fifth millimeter when the optical parameters are modified to reflect the new wavelength.

Figure 12 shows the case where photons inelastically scatter into the 1582 cm^−1^ bin. When the optical parameters are modified to reflect the new wavelength, the number of photons exiting the model increases by 18% in the first millimeter (from the excitation point) to 13% by the fifth millimeter away from the light source.

**Figure 13:**
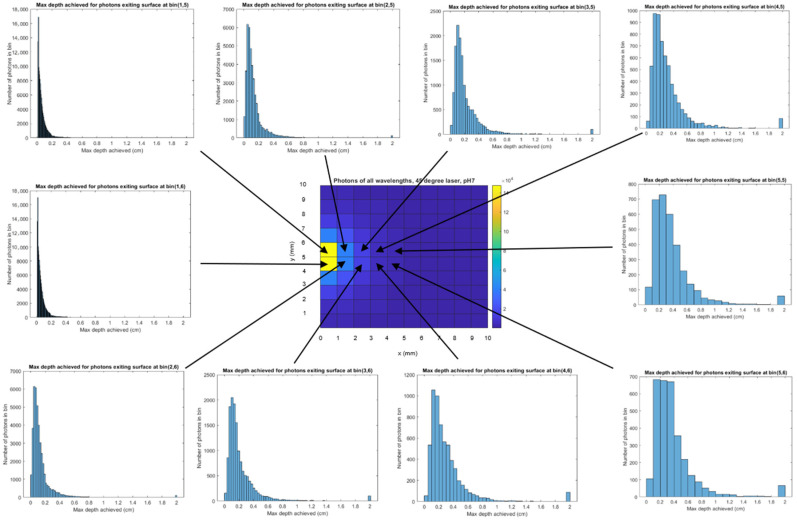
For the leftmost column of the tissue model’s horizontal exit plane, the histograms of depth profiles vs. number of photons are shown. As the number of photons increases (near the excitation at the center of column 1 and between row 5 and 6), the depth shifts left (smaller).

In Figure 13, we combine photons of all wavelengths to create histograms of the maximum depth achieved by each photon during its trajectory of interactions in relation to the exit point of the photon on the model surface. Associated with the 0 mm offset, the peak number of photons (~18 K) exiting the 1 × 1 mm area has achieved a depth of ~0 mm (Figure 13 upper left), while at the 5 mm offset, the peak number of photons (~700), has achieved a depth of 3 mm. The modeling results exhibit several phenomena. Most importantly, an increase in the maximum depth achieved by a photon is associated with an increase in the photon’s distance from the excitation upon its exit (Figure 13), which matches the SORS principle [3]. However, the number of photons exiting the top of the model is proportional to the proximity to the excitation point (Figure 11, Figure 12 and Figure 13). This is shown via: the heatmap color (Figure 11, Figure 12 and Figure 13), the number in each grid square (Figure 11 and Figure 12 for the two MBA reference peaks) and the declining number of photons of all wavelengths (Figure 13’s histograms) as offset increases. Therefore, the number of photons in each bin (or grid square) is not proportional to an increase in maximum depth; the histograms with a high number of photons have a shallower depth associated with their peak number of photons. Finally, it should be noted that prevalence of photons with a 2 cm depth (the rightmost bar in Figure 13’s histograms) is an artifact of the boundary condition that is set to allow photons to leave if they reach a boundary (i.e., all the photons that go past the final bin are constrained to the last bin).

In summary, the model results show that the strongest Raman signals are nearer to the excitation than expected, and therefore, 0 and 1 mm detector offsets should be included when sweeping the area to locate an implant’s maximum signal.

### 3.3. Comparison of Experimental and Modeling Results

**Figure 14:**
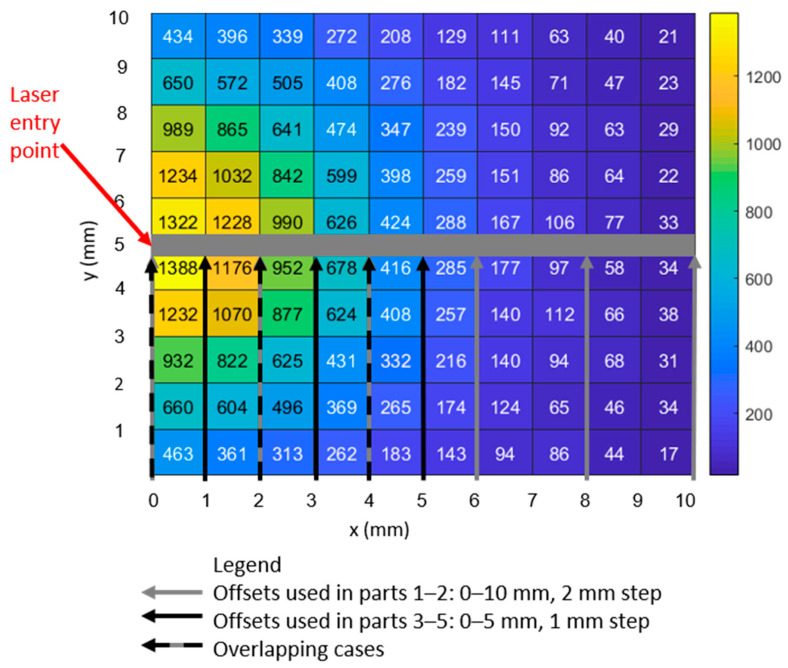
Overlaying the experimental offsets onto the model’s surface. This figure is based on Figure 11b, the 1072 cm^−1^ reference peak, with the model grid overlaid upon it.

The experimental and simulated layouts are superimposed in Figure 14 to illustrate the commonality of the geometries. The solid black arrows indicate the points of detection used to create the plots in Figure 11 and Figure 12.

The 3D tissue model positions the ~1 mm thick SERS-active target at a depth ranging from 2 to 3 mm; this depth corresponds to the experimental setups with a 2 mm thick barrier, which are found in Figure 7c and Figure 9c. The model maps closest to the 2 mm thick barrier case (i.e., experimental Parts 1c, 2c, 3c, 4c and 5c). The intensities of both reference peaks expected to be brightest at 0 offset, with steep linear decline to the max offset (i.e., 10 mm in Parts 1–3 and 5 mm in Parts 4–5).

Considering the intensities of the 1072 cm^−1^ peak values (blue), it appears that the degradation of the signal matches the decline in the number of photons at the same positions in the heatmap in Figure 11. However, calculating the relative percentage losses, the experimental values are exceeded by the model. Specifically, the experimental results decline in intensity from 0 to 5 mm offset, by 40% and 20%, respectively (1072 cm^−1^ peak values, blue, in Figure 7c and Figure 9c). In contrast, the model displays losses of 69% (calculated via averaging the number of photons with 1072 cm^−1^ Raman shift in heatmap bins bounded by y = 4–6 mm, over the same range of 0–5 mm offsets in Figure 11).

This discrepancy could be caused by differences between the tissue model and the experimental tissue used. The model consists of dermal and epidermal layers which were not represented in the experimental setups. To obtain the precise range of thicknesses needed for the experimental setups, only porcine muscle tissue could be used. While there was an attempt made to use porcine tissue with its skin for this study, this was found unsuitable because of surface irregularities and a corresponding difficulty to slice it to uniform thicknesses parallel to the surface. Since a future goal is to perform actual in vivo sensing of implanted SERS-active hydrogels, the tissue model matches in vivo conditions as closely as possible.

**Figure 15:**
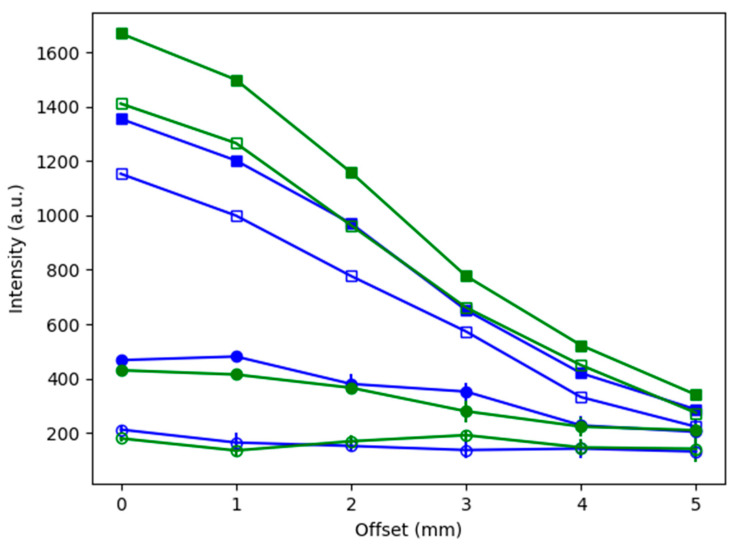
Additional comparison of experimental and model results. All for the 2 mm thick barrier. Legend: symbols indicate the dataset: filled circles = experimental contiguous barrier; hollow circles = experimental stratified barrier; hollow squares = model with unchanged optical properties; filled squares = model with changed optical properties after Raman scattering. Colors indicate the MBA peak: blue = 1072 cm^−1^ peak, green = 1582 cm^−1^ peak. N = 15 for each experimental data point, while the model data are from a single run with millions of photons.

An additional comparison of the experimental and modeling results, including a representation of the effect of changing the optical properties after Raman scattering, is presented in Figure 15. The intensities of the two MBA reference peaks for the experimental cases from Part 4c and 5c (contiguous and stratified, respectively) are plotted alongside those from the model. These experimental datasets were selected because their 2 mm barrier thickness matches the model. Note that the modeled intensities are given by the number of photons (averaged for the bins above and below the line, y = 5 (as shown by the thick gray line in Figure 14).

Considering Figure 15, all the datasets (both experimental and simulated) are seen to share the trend of diminishing intensity with increasing offset and that there is little evidence of the SORS effect. This means that, with the possible exception of the 1072 cm^−1^ peak for Part 4c (blue squares) at offset = 1 mm, none of the signals increase as the offset increases from 0 mm. Note that, as previously stated, the fact that the experimental intensities are in arbitrary units and that the model intensities are represented by the number of photons (exiting from that location of the grid) means that only the trends in the datasets can be compared. However, it is possible to measure the effect of changing the optical properties after Raman scattering by comparing the increase in intensities from the “hollow squares” dataset to the “solid squares” dataset for both the 1072 and 1582 cm^−1^ peaks (blue and green, respectively). The increase in the intensity of the data when the optical properties change is seen for all values (solid squares > hollow squares) and for both MBA peaks. Specifically, the intensities for the 1072 cm^−1^ peak (blue), using the modified Raman scattered optical properties (solid squares) show increases of 17.5, 20.2, 24.9, 13.7, 26.5 and 28.5% for offsets 0 through 5 mm, respectively, relative to the intensities resulting when the optical properties are constant (hollow squares). Similarly, for the 1582 cm^−1^ peak (green), the increases in intensity are 15.4, 15.5, 16.8, 14.9, 14.0 and 19.5% for offsets 0 through 5 mm, respectively.

If these percentage increases in the intensity of the two MBA reference peaks are caused by the decreases in the scattering coefficients with increasing wavelength (Figure 4), then the magnitudes of the changes should be related. The comparison is complicated by the fact the increases in intensity are given over a range of offsets, while the scattering coefficients vary by tissue type. In addition, the scattering coefficient is the inverse of the mean free path. Focusing on the 0 mm offset intensity and the dermis tissue type, the two MBA reference peaks show these changes: the 1072 cm^−1^ peak’s scattering coefficient decreases by 14.68% while the peak intensity increases by 17.5%. Similarly, the 1582 cm^−1^ peak decreases by 15.4% while the peak intensity increases by 20.9%. The magnitudes of the changes in the scattering coefficient and intensity show evidence of causality, for the cases (0 mm offset and dermis tissue type) that were selected.

## 4. Discussion

This work has sought to overcome some of the limitations in the SORS literature concerning the application of SORS to the in vivo detection of implanted SERS-active targets. First, regarding the target size: for a target the size of an implant, these results differ from those found in the SORS literature where the target is typically as extensive as the barrier (in the plane parallel to the sensing surface). While the MBA reference peaks were detected to have, at best, the same intensities at offsets of 1 and 2 mm under a 2 mm thick barrier compared to the zero-offset case, no increase in intensity with offset was detected. This differs from the typical SORS case. Second, considering the presence (or absence) of a volume of turbid media under the target: the addition of a layer of tissue under the SERS-active target served to retain 60% more of the signal when the target was at a depth of 1 mm. While an improvement in signal strength was expected due to the increased volume of turbid media available to the photons for scattering, this is the first time that it has been quantified. Third, regarding the continuity of the barrier layer over the target: the difference between using a contiguous barrier and a stratified barrier on top of the target was a 50% signal intensity loss as well as a loss of distinction across the four MBA peaks, even for the thinnest case where two 1 mm thick layers were compared to one 2 mm thick layer. There was further incremental degradation in the three and four 1 mm thick-layer cases of 20% and 25%, respectively. While increasing losses with barrier thickness were anticipated, to our knowledge this is the first time that they have been quantified.

Regarding the model, there is a large (13–30%, depending on distance from excitation) increase in the number of photons exiting from the upper surface when the optical parameters are modified to reflect the new Stokes-shifted wavelength after inelastic scattering. This increase reveals that, when light-tissue interaction models use constant optical properties, Raman signal intensities are underestimated. This makes sense because the Raman shift is to longer wavelengths, which typically correspond to longer mean free paths (1/µ_s_). And while the tissue model was not a direct match to the tissue used in the experimental model, the spatial distribution of the exit position of the photons (which have interacted with the implant and changed their wavelength) is shown to exhibit the same trend, i.e., the number of photons is greatest at the zero-offset position and declines with offset. All of the above data are helpful to define the parameters to use when measuring in vivo implanted SERS-active targets.

## Figures and Tables

**Table 1:** Layout of the SORS experimental conditions. Barrier = porcine muscle tissue barrier is present/absent. Thickness = if barrier present, thickness of barrier. Type = barrier is contiguous (one piece) or stratified (made of 1 mm layers). Offset = Range of SORS offsets. Step = SORS offset step size. Base = base under target is either none or 5 mm thick porcine tissue slab.

Part	Barrier	Thickness (mm)	Type	Offset (mm)	Step (mm)	Base
1a	absent	N/A	N/A	0	N/A	none
1b	present	1	contiguous	0–10	2	none
1c	present	2	contiguous	0–10	2	none
1d	present	3	contiguous	0–10	2	none
2a	absent	N/A	N/A	0–10	N/A	5 mm thick
2b	present	1	contiguous	0–10	2	5 mm thick
2c	present	2	contiguous	0–10	2	5 mm thick
2d	present	3	contiguous	0–10	2	5 mm thick
3a	absent	N/A	N/A	0–10	N/A	5 mm thick
3b	present	1	contiguous	0–10	2	5 mm thick
3c	present	2	contiguous	0–10	2	5 mm thick
3d	present	3	contiguous	0–10	2	5 mm thick
3e	present	4	contiguous	0–10	2	5 mm thick
4a	absent	N/A	N/A	0	N/A	5 mm thick
4b	present	1	contiguous	0–5	1	5 mm thick
4c	present	2	contiguous	0–5	1	5 mm thick
4d	present	3	contiguous	0–5	1	5 mm thick
4e	present	4	contiguous	0–5	1	5 mm thick
5a	absent	N/A	N/A	0	N/A	5 mm thick
5b	present	1	stratified	0–5	1	5 mm thick
5c	present	2	stratified	0–5	1	5 mm thick
5d	present	3	stratified	0–5	1	5 mm thick
5e	present	4	stratified	0–5	1	5 mm thick

## Data Availability

The data presented in this study are available upon request from the corresponding author. The data are not publicly available due to privacy.

## Supplementary Materials

The supporting information can be downloaded at: https://www.mdpi.com/article/10.3390/bios14020081/s1.
