# Supplementary material for: Surface-Enhanced Spatially Offset Raman Spectroscopy in Tissue
Source: Biosensors (Basel). 2024 Feb 2;14(2):81. doi: 10.3390/bios14020081 (PMC10886963; doi:10.3390/bios14020081)
Supplement: Supplementary file 1 [file biosensors-14-00081-s001.zip › biosensors-2792535-supplementary.pdf]

biosensors

Supplementary Information for Article

Surface-enhanced spatially offset Raman spectroscopy in tissue

Dayle Kotturi [1], Sureyya Paterson [1] and Mike McShane [1,2,*]

[1] Texas A&M University, Department of Biomedical Engineering, College Station, Texas, United States
[2] Texas A&M University, Department of Materials Science and Engineering, College Station, Texas, United States
* Correspondence: mcshane@tamu.edu; Tel.: 1 979 845-7941; fax 1 979 845-4450; biosym.org

Citation: Kotturi, D.; Paterson, S.; McShane, M. Surface-Enhanced Spatially-Offset Raman Spectroscopy in Tissue. *Biosensors* **2024**, *14*, x. https://doi.org/10.3390/xxxxx

Received: 19 December 2023
Revised: 22 January 2024
Accepted: 24 January 2024
Published: 2 February 2024

Copyright: © 2024 by the authors. Submitted for possible open access publication under the terms and conditions of the Creative Commons Attribution (CC BY) license (https://creativecommons.org/licenses/by/4.0/).

1. Modeling

To start with, the public software found at https://omlc.org/software/mc/ was modified to the version found in https://github.com/karendayle/monteCarlo, which is a public repository. The software modifications are described in detail as:

- Start with OMLC.org's light-tissue interaction code (files are: mcxyz.c and MATLAB® 2019 scripts: makeTissue.m and makeTissueList.m)
- Modify mcxyz.c as follows:
    - Introduce the probability of a Raman inelastic scattering event inside the scattering code. Make it large enough for the model to work and then normalize to reflect the accepted Raman probability reality factor when done.
    - Set up a 2D array of optical parameters where the row dimension contains the wavelengths (nm) of the target Raman peaks (using Table S-1 conversion). The original code allowed only one wavelength and determined the optical parameters based on that. The resulting set of optical parameters are shown in Table S-2 through Table S-6.
    - Set up a probability table (Table S-7) which expresses the area under the Raman spectrum of an MBA peak of interest divided by the total area under the entire MBA Raman spectrum. Use this to assign the outcome of the inelastic event. Note that since the MBA spectrum is affected by environmental pH level, the values in the table can be altered to reflect this change.
    - Set a variable to flag that a photon was inelastically scattered and do not allow it to reoccur for the current photon, although keep track of the number of attempted repeat events.
    - Update the optical parameters that are used to be those based on the new wavelength for the duration of the current photon's trajectory.
    - As the photon moves through the tissue structure, keep track of: 1) how deep it goes and 2) the x,y coordinates of its final position if it exits through the top surface.
    - Make heat maps of the final photon position and histograms of the photon population for various conditions.
- Modify the makeTissue.m and makeTissueList.m as follows:
    - Add new tissue type for SERS-active hydrogel to the list
    - Define a 3D disc shape in the tissue model to match the 6 mm diameter disc shape of the actual target used in Parts 1-5 and add it to the drawing of both front and side views of tissue model
- Write new code to create heatmaps and histograms to display results
- Additional modifications:
    - Upon inelastic scatter event, update the optical coefficients for all tissue types to carry the photon back through the structure. This means that, to

see the effect of the new wavelength, look in the dermis and epidermis (which have new wavelength-dependent optical properties)
- Change probability weightings so there is no "catchall" bin since this case does not match a single wavelength, but rather all the wavelengths that are not targets
- Fix up the array starting at 0 case, since there is MATLAB® 2019 code that runs after the c code. In c, arrays start at index 0; in MATLAB® 2019, they start at index 1
- Limit the model to only 1 pH level at a time, or it would be necessary to change the muav, musv and gv arrays to 3D

Table S-1. Conversion of MBA peak wavenumbers to wavelength and the closest matching COTS BPFs, based on an excitation wavelength of 785 nm. WL = wavelength when excitation is 785 nm. Ideal = Ideal narrow BPF's CWL. Best = closest COTS BPF's CWL.

Target	WN [cm^{-1}]	WL [nm]	Ideal [nm]	Best [nm]
MBA ref peak 1	1072	857.1	857	860
MBA pH peak 1	1430	884.3	884	880
MBA ref peak 2	1582	896.3	896	900
MBA pH peak 2	1702	906.0	906	910

The optical properties for the wavelengths of interest are given in the next five tables.

Table S-2. Values for excitation wavelength.

Fields	Name	μa	μs	g
1	Air	0.0001	1	1
2	Water	0.0224	10	1
3	Blood	4.2827	63.6943	0.9000
4	Dermis	0.0233	145.8914	0.9000
5	Epidermis	4.5536	254.7771	0.9000
6	Skull	0.0100	191.0828	0.9000
7	Gray matter	0.0594	108.4944	0.9000
8	White matter	0.0594	108.4944	0.9000
9	Standard tissue	1	100	0.9000
10	SERS-active hydrogel	1	100	0.9000

Table S-3. Values for ref peak 1 wavelength.

Fields	Name	μa	μs	g
1	Air	0.0001	1	1
2	Water	0.0456	10	1
3	Blood	5.2969	58.3431	0.9000
4	Dermis	0.0398	124.4612	0.9000
5	Epidermis	3.4214	233.3722	0.9000
6	Skull	0.0186	175.0292	0.9000
7	Gray matter	0.0868	97.9835	0.9000
8	White matter	0.0868	97.9835	0.9000
9	Standard tissue	1	100	0.9000
10	SERS-active hydrogel	1	100	0.9000

Table S-4. Values pH peak 1 wavelength.

Fields	Name	μa	μs	g
1	Air	0.0001	1	1
2	Water	0.0578	10	1
3	Blood	5.7160	56.5611	0.9000
4	Dermis	0.0485	118.0359	0.9000
5	Epidermis	3.0981	226.2443	0.9000
6	Skull	0.0230	169.6833	0.9000
7	Gray matter	0.0999	94.5916	0.9000
8	White matter	0.0999	94.5916	0.9000
9	Standard tissue	1	100	0.9000
10	SERS-active hydrogel	1	100	0.9000

Table S-5. Values pH peak 1 wavelength.

Fields	Name	μa	μs	g
1	Air	0.0001	1	1
2	Water	0.0650	10	1
3	Blood	5.8510	55.8036	0.9000
4	Dermis	0.0534	115.4028	0.9000
5	Epidermis	2.9694	223.2143	0.9000
6	Skull	0.0256	167.4107	0.9000
7	Gray matter	0.1066	93.1646	0.9000
8	White matter	0.1066	93.1646	0.9000
9	Standard tissue	1	100	0.9000
10	SERS-active hydrogel	1	100	0.9000

Table S-6. Values for pH peak 2 wavelength.

Fields	Name	μa	μs	g
1	Air	0.0001	1	1
2	Water	0.0709	10	1
3	Blood	5.9543	55.1876	0.9000
4	Dermis	0.0575	113.3034	0.9000
5	Epidermis	2.8679	220.7506	0.9000
6	Skull	0.0278	165.5629	0.9000

7	Gray matter	0.1121	92.0107	0.9000
8	White matter	0.1121	92.0107	0.9000
9	Standard tissue	1	100	0.9000
10	SERS-active hydrogel	1	100	0.9000

Table S-7. Given an inelastic scattering event, this table shows the probabilities that the new wavelength will match the peaks of interest, based on the area under the MBA spectrum at pH 7.0, which has been approximated as 56 square arbitrary units. The assumption is that, given the event, one of four outcomes will occur. This decision was made in order to be able to compute the optical properties for each wavelength. Having a catchall bin to contain all the wavelengths that do not match any of the peaks of interest (as in previous version) became impossible since the optical parameters are based on a single wavelength. WL = wavelength. WN = wavenumber.

Target	WL index	WN [cm^{-1}]	pH 7.0 probability	Bin range
N/A	0	excitation	N/A	N/A
0	1	1072	21/56	0.000 <= x < 0.375
1	2	1430	6/56	0.375 <= x < 0.482
2	3	1582	26/56	0.482 <= x < 0.946
3	4	1702	3/56	0.946 <= x < 1.000

2. Results

Parts 1 - 3 describe SORS experiments using a 6 mm diameter, 1 mm thick disc of SERS-active pHEMA hydrogel, first uncovered with 0 mm offset and then through different thicknesses of tissue (barrier layer) for a range of offsets and incremental step size as listed in each heading.

2.1 Part 1: 1, 2 and 3-mm barriers, 0- through 10-mm offsets

Figure S-1a shows the maximum intensity of the MBA reference peaks as ~10,000 arbitrary units (a.u.). In Figure S-1b, the 1 mm thick barrier layer allows 7% of the maximum intensity (700/10,000) with only the 1 mm thick barrier layer and no offset. As the offset increases, the intensity drops off by another half (600 to 300 a.u.) although the 1072 cm^{-1} peak does show a higher value at an offset of 6 mm. In Figure S-1c, the 2 mm thick barrier layer shows a linear decline in intensity in both of the reference peaks as the offset ranges from 0 to 6 mm. In Figure S-1d, the 3 mm thick barrier layer shows the only instance where the signal strength increases with offset, but only for the 1072 cm^{-1} peak.

These results do not show the expected increase in intensity with offset for any of the barrier layer thicknesses.

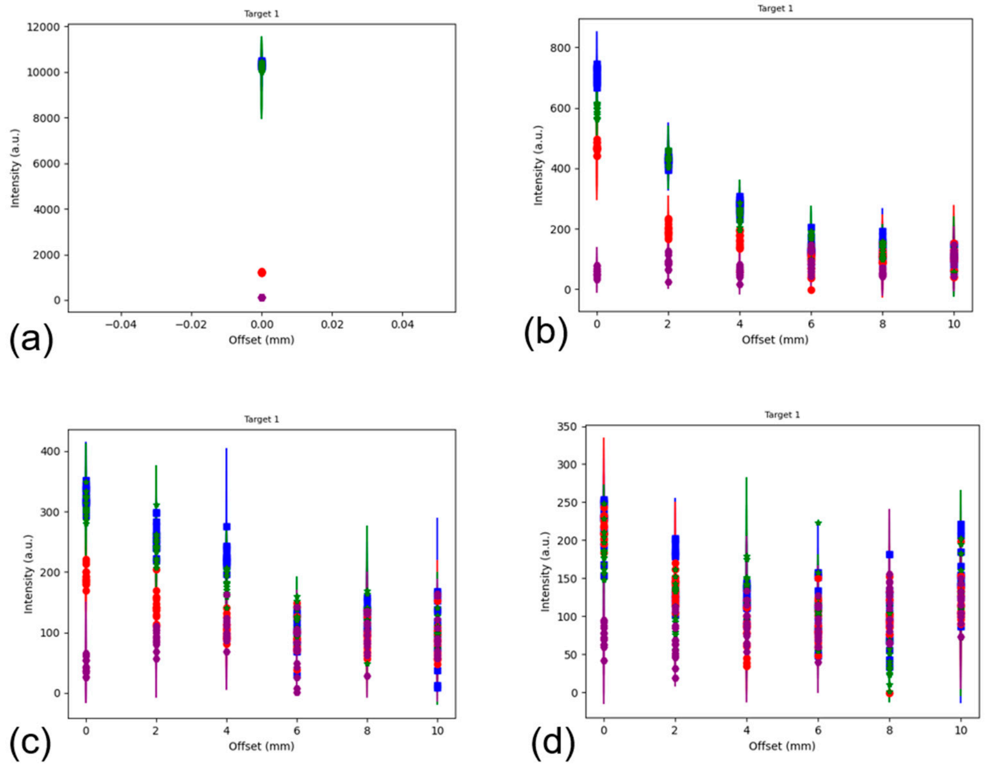

Figure S-1. Raman scattering intensities of hydrogel discs as measured in four different experimental setups. (a) uncovered disc, (b) disc under 1 mm tissue, (c) disc under 2-mm tissue, (d) disc under 3-mm tissue. Legend: blue = 1072 cm^{-1} peak, green = 1582 cm^{-1} peak, red = 1430 cm^{-1} peak, purple = 1702 cm^{-1} peak. N = 15. Circle is mean, error bars are ± 1 std dev. Integration time = 2000 ms, laser output power = 66 mW.

In Part 1, it was found to be more difficult to align the 0 mm offset position at the start when the target is covered (compared to when the target is uncovered). With the barrier layer in place, the laser appears to move to the right of the target. As the barrier layer thickness increases, the laser shifts further right (Figure S-2) This alignment approach was used for all barrier layer thicknesses. It was decided for all measurements in this study that the laser would align with the uncovered target. This is an issue which must be considered when the target is in vivo. In the future case of actual in vivo sensing, there will be no way to align the laser and detector over the uncovered target. The optimal alignment will look “off” when viewed from the outside.

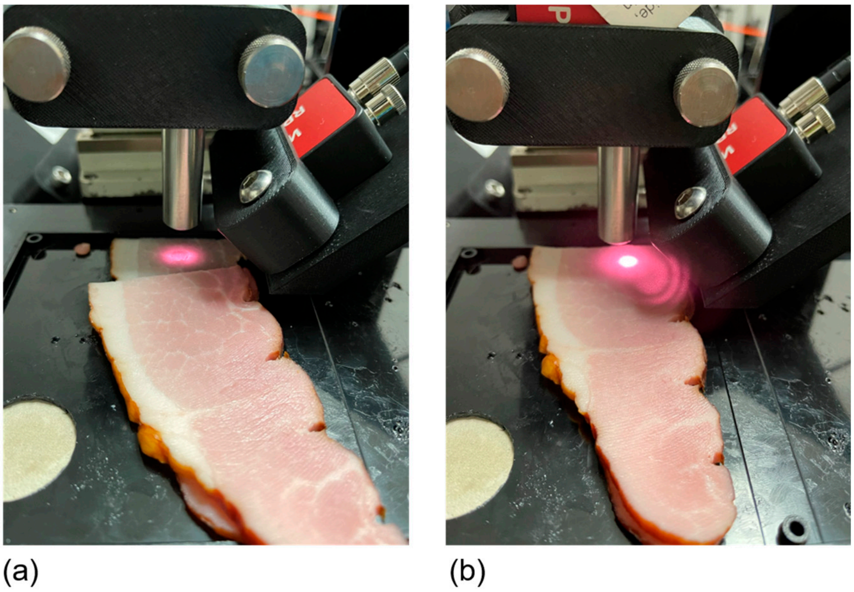

Figure S-2. Shows how the laser beam moves right when barrier layer is applied. (a) barrier layer is removed to expose the alignment of laser (right probe with barrel removed). (b) barrier layer is in place. The laser beam moves to the right because of the additional height. The position of both probes is unchanged between the left and right photos.

Other SORS studies explore ideas such as investigating the importance of the point of focus. For example, one study looks at doing SORS with a single probe provided that the laser is focused at the target depth.[1] This is an important point that is rarely mentioned but which was critical to the work presented herein. Another is the first study to remove the focusing optics of the laser probe and work with a diffuse laser beam in order not to not limit the laser light to a single point of focus in the tissue.[2]

2.2 Part 2: 1, 2 and 3 mm barriers, on tissue base, 0 through 10 mm offsets

The importance of having a 3D space of turbid media under the target is known.[2] By adding it in Part 2, the expected increase in the Raman peak intensities compared to Part 1 is tested.

With the addition of the 5 mm thick base under the target, there is an increase in the peak intensities of the reference peaks. Here, with an uncovered maximum of ~8000 a.u. (Figure S-3a), the 1 mm thick-barrier layer reduces the intensity to ~900 a.u. at zero offset. This reduced intensity is 11.2% of the maximum and more than twice the value when there is no base present (see Part 1). As the barrier layer thickness increases from 1 mm to 3 mm (Figure S-3B through Figure S-3d), the intensity of both reference peaks shows a linear decline in intensity. Figure S-3b, the 1 mm thick barrier layer, shows a linear decline to an offset of 6 mm; Figure S-3c, the 2 mm thick barrier layer, shows a linear decline to an offset of 8 mm; and Figure S-3d, the 3 mm thick barrier layer, shows a linear decline which is steeper for the 1072 cm^{-1} case (blue) than the 1582 cm^{-1} case (green) to an offset of 4 mm.

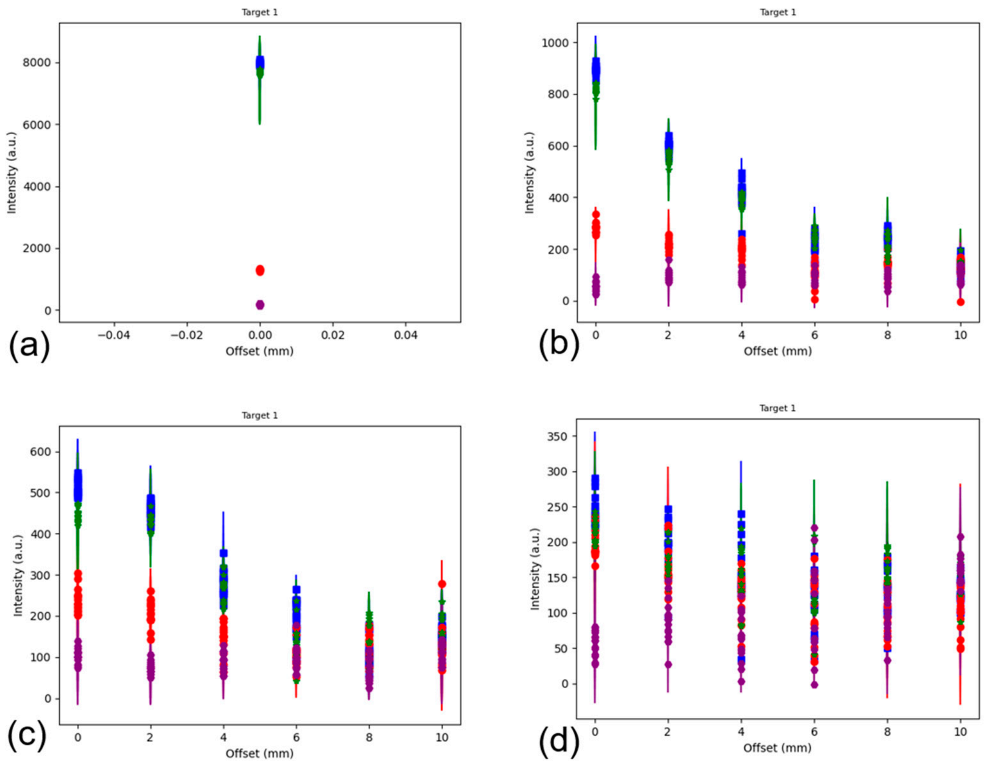

Figure S-3. Raman scattering intensities of hydrogel discs as measured in four different experimental setups. (a) uncovered disc, (b) disc under 1-mm tissue, (c) disc under 2-mm tissue, (d) disc under 3-mm tissue, all on 5 mm solid base. Legend: blue = 1072 cm^{-1} peak, green = 1582 cm^{-1} peak, red = 1430 cm^{-1} peak, purple = 1702 cm^{-1} peak. N = 15. Circle is mean, error bars are ± 1 std dev. Integration time = 2000 ms, laser output power = 66 mW.

2.3 Part 3: 1, 2 and 3 mm barriers, on tissue base, 0 through 5 mm offsets

Part 3 narrows the offset and step size to focus on how the intensity changes in the first 5 mm. Figure S-4a shows the maximum intensity as ~9000 a.u. of the two reference peaks (blue and green). When the 1 mm barrier layer is applied (Figure S-4b), the signal is degraded to less than 10% of the maximum. However, the linear decline in intensity does not begin until the offset is 3 mm; i.e., the reference peak intensities remain approximately constant for offsets = 0, 1 and 2 mm. This behavior is repeated in Figure S-4c where the barrier layer is 2 mm thick. In Figure S-4d, the intensities are small overall, but appear largest when the offset is 1 mm.

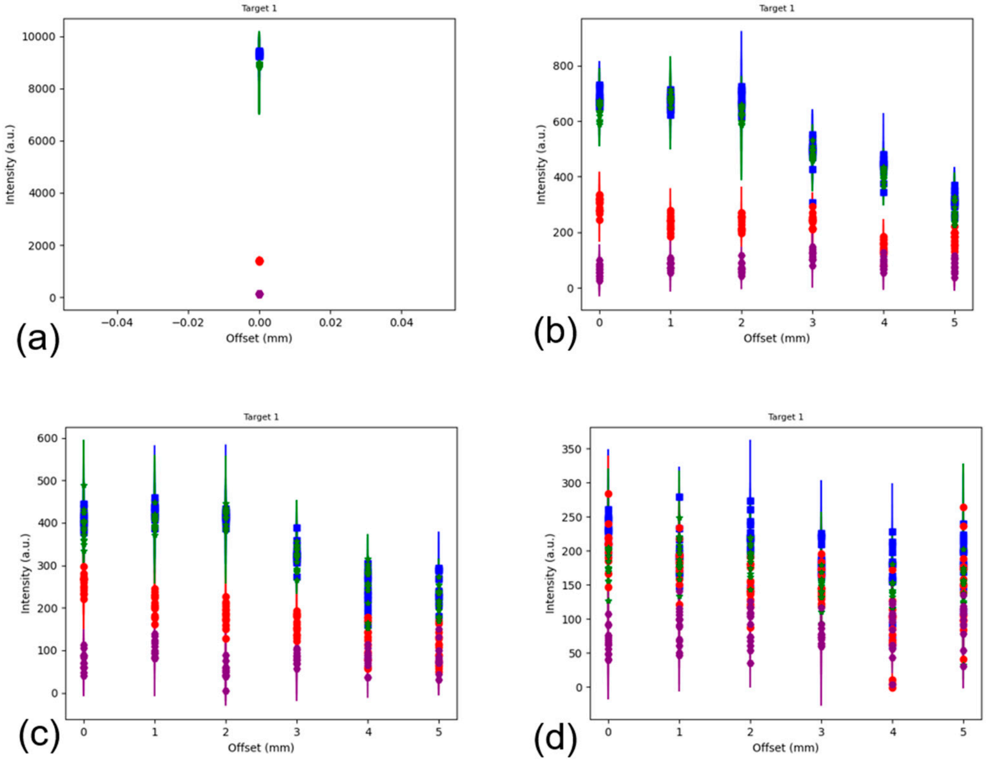

Figure S-4. Raman scattering intensities of hydrogel discs as measured in four different experimental setups. (a) uncovered disc. (b) disc under 1 mm tissue. (c) disc under 2 mm tissue. (d) disc under 3 mm tissue, all on 5 mm solid porcine muscle tissue base. Legend: blue = 1072 cm^{-1} peak, green = 1582 cm^{-1} peak, red = 1430 cm^{-1} peak, purple = 1702 cm^{-1} peak. N = 15. Circle is mean, error bars are ± 1 std dev. Integration time = 2000 ms, laser output power = 66 mW.

References

1. Asiala, S.M., et al., *Surface-Enhanced, Spatially Offset Raman Spectroscopy (SESORS) in Tissue Analogues.* ACS Appl Mater Interfaces, 2017. **9**(30): p. 25488-25494.
2. Nicolson, F., et al., *Non-invasive In Vivo Imaging of Cancer Using Surface-Enhanced Spatially Offset Raman Spectroscopy (SESORS).* Theranostics, 2019. **9**(20): p. 5899-5913.
